# Effects of maximum residue limit of triflumezopyrim exposure on fitness of the red imported fire ant *Solenopsis invicta*

**DOI:** 10.7717/peerj.8241

**Published:** 2019-12-10

**Authors:** Qiting Li, Fei Zhao, Jiayi Li, QiuHong Tao, JiaQian Gao, Yong-Yue Lu, Lei Wang

**Affiliations:** 1 College of Agriculture, South China Agricultural University, Guangzhou, Guangdong, China; 2 Guangdong Tianhe Agricultural Means of Production Co., Ltd., Guangzhou, Guangdong, China

**Keywords:** Triflumezopyrim, Fire ant, nAChR, Aggressiveness, Colony growth, Behavior

## Abstract

The impact of exposure to free feeding concentrations of triflumezopyrim to the red imported fire ant, *Solenopsis invicta*, in maximum residue tolerances for 56 days was investigated to understand whether triflumezopyrim, a novel neonicotinoid, poses unacceptable risks to the environment. Our results demonstrated that neither 0.5 μg/ml nor 0.2 μg/ml triflumezopyrim have a significant impact on the growth of the *S. invicta* colony and their food consumption (sugar water and locusts) during the length of treatment. While both 0.5 μg/ml and 0.2 μg/ml triflumezopyrim improved the grasping ability of *S. invicta*, and 0.5 μg/ml not 0.2 μg/ml triflumezopyrim increased their rate of locomotion. In addition, although 0.5 μg/ml and 0.2 μg/ml triflumezopyrim increased their individual aggressiveness index, the probability of the survival of *S. invicta* was not impacted by triflumezopyrim treatments in aggressive group encounters. This study suggests that triflumezopyrim did not have a negative impact on the fitness of *S. invicta* at 0.5 μg/ml and 0.2 μg/ml exposures.

## Introduction

Neonicotinoids are insecticides that act on the nicotinic acetylcholine receptor of insects; it exhibits good activity on sent hopper controap-sucking pests and comprises over 30% of insecticide sales in the world (Hladik, Main & Goulson, 2018; Ihara & Matsuda, 2018). However, the negative impact of neonicotinoids on non-target organisms at their sublethal concentrations is of increasing concern (Butcherine et al., 2019; Wang et al., 2018). Research has shown that neonicotinoids have adverse impacts on pollinator insects, especially honeybees, and three types of this chemical are currently banned from the European Union, including imidacloprid, thiamethoxam and clothianidin (Ihara & Matsuda, 2018; Pisa et al., 2015).

Current, novel neonicotinoids were developed including sulfoxaflor, flupyradifurone and triflumezopyrim. Researchers have considered the safety of these new neonicotinoids over other neonicotinoids for non-target insects, especially honeybees. Pan, Lu & Wang (2017) found that 1.0 μg/ml sulfoxaflor reduced aggressiveness, colony growth, and food consumption of the fire ant *Solenopsis invicta*, while Siviter et al. (2019) found that acute sulfoxaflor exposure did not have negative impacts the olfactory conditioning and working memory of bees. Research showed that flupyradifurone impairs olfactory learning, motor abilities, and the survival of honeybees, including *Apis mellifera carnica*, *A. mellifera ligustica*, and *A. cerana* at even field-realistic doses (Hesselbach & Scheiner, 2019; Tan et al., 2017; Tosi & Nieh, 2019).

Triflumezopyrim is developed by DuPont Crop Protection and is effective against sap-sucking pests (Cordova et al., 2016; Zhang, 2017; Zhang et al., 2017). It was registered in China, Korea, and the Philippines in 2016 and Pyraxalt™ 10% triflumezopyrim SC, the commercial product, was brought to market in China in 2017 (http://news.agropages.com/News/NewsDetail---19109.htm, access on 2019-6-2; http://www.agroinfo.com.cn/other_detail_4668.html, access on 2019-6-2). Zhu et al. (2018) found that triflumezopyrim is an effective insecticide for suppressing plant hopper populations and is harmless to their natural enemies including *Anagrus nilaparvatae*, *Cyrtorhinus lividipennis*, *Paederus fuscipes*, *Pirata subpiraticus*, *Ummeliata insecticeps*, *Hylyphantes graminicola*, and *Pardosa pseudoannulata*. Since sulfoxaflor and flupyradifurone were reported to have negative effects on non-target insects, the effects of triflumezopyrim on non-target insects deserves greater attention.

Ants are an important species and a crucial member of their ecosystem; their disruption would negatively impact the health of the ecosystem (Toro, Ribbons & Pelini, 2012). Imidacloprid can change the foraging and competitive behaviors of *Lasius niger* and *L. flavus* at sublethal concentrations, which may alter the structure and dynamics of ant communities after oral treatment (Thiel & Köhler, 2016). One μg/ml imidacloprid oral treatment reduced the aggressive behavior of the native ant, *Monomorium antarcticum*, to the invasive ant, *Linepithema humile*, which may facilitate a successful invasion by the invasive species (Barbieri et al., 2013). Ants are important predators and terrestrial herbivores with a large amount of soil contact and were exposed to neonicotinoids by oral exposure through plant ingestion as well as by contact exposure through contaminated soil (Hölldobler & Wilson, 1990).

The red imported fire ant, *S. invicta*, is a well-studied ant species making it an ideal model on which to study the sublethal effects of neonicotinoids on ants (Vinson, 2013; Wurm et al., 2011). *S. invicta* is an active and efficient predator of pests in agroecosystems despite its negative effects on public health, agriculture and biodiversity (Harvey & Eubanks, 2004; Vogt et al., 2001; Wang et al., 2016). In this study, we exposed *S. invicta* colonies to residue limits of triflumezopyrim and evaluated the impact of exposure on their fitness. The objective of the study was to determine the effects of the maximum residue limit of triflumezopyrim on the fitness of the colony and the behavior of workers.

## Materials and Methods

### Insects and insecticide

Red imported fire ants, *Solenopsis invicta*, were collected from an organic farm in Conghua County, Guangzhou, China and reared at the Red Imported Fire Ant Research Centre at the South China Agricultural University. *S. invicta* colonies were provided with a 10% w/w sugar water solution and frozen locusts *Locusta migratoria*. The social form of *S. invicta* colonies were determined using methods of Shoemaker & Ascunce (2010), and only polygyne colonies were used in this experiment. All bioassays were conducted under laboratory conditions, and all colonies were reared in the laboratory at least 1 week prior to the start of the experiment.

The commercially available insecticide, Pyraxalt™ 10% triflumezopyrim SC (DuPont™ Crop Protection, Pudong, Shanghai, China), was used in all experiments.

### Effect of triflumezopyrim on *S. invicta* food consumption and colony growth

The Environmental Protection Agency (EPA) of the United States establishes tolerances for the residues of triflumezopyrim in rice grains and rice hulls of 0.4 ppm and 1.0 ppm, respectively (https://www.federalregister.gov/documents/2017/10/16/2017-22356/triflumezopyrim-pesticide-tolerances, accessed on 2019-6-4). Therefore, we used 0.5 and 0.2 μg triflumezopyrim/ml for the residue tolerances of triflumezopyrim based on the regulatory requirements of the EPA.

Pyraxalt™ is advertised to provide up to 25 days of consistent hopper control (http://www.dupont.com/products-and-services/crop-protection/pyraxalt-insect-control.html, access on 2019-6-2). The estimated average life span of minor, medium, and major workers of *S. invicta* is 45 days, 75 days and 135 days, respectively (Calabi & Porter, 1989). In light of these two combined factors the experiment was conducted over 8 weeks.

The experimental method was reflective of the work of Pan, Lu & Wang (2017) in which a colony was divided into the same weight (>20 g), and each sub-colony contained workers, brood, larva, pupa, and at least one functional queen. Each sub-colony then received either the control 10% w/w sugar water or a single concentration of triflumezopyrim (0.2 μg/ml or 0.5 μg/ml) in 10% w/w sugar water; solutions were provided in a 15 ml glass tubes with a cotton stopple. Sub-colonies received a sufficient amount of locusts. The weight of each sub-colony was taken every 7 days to evaluate the effect of triflumezopyrim on colony growth of *S. invicta*. The accumulated percentage weight loss of each sub-colony was calculated after 7 days, 14 days, 21 days, 28 days, 35 days, 42 days, 49 days and 56 days of treatment (Pan, Lu & Wang, 2017). Sugar water and locust consumption (mg sugar water or locust per g ant) was also calculated for each sub-colony at 1 day, 7 days, 14 days, 21 days, 28 days, 35 days, 42 days, 49 days and 56 days. Water evaporation was also estimated. Four colonies were used in this experiment.

### The effect of triflumezopyrim on active behavior of *S. invicta*

We evaluated the effect of triflumezopyrim on *S. invicta* behavior by measuring their grasping ability and walking speed after 56 days of treatment. The experiment method was reflective of the method used by Huang et al. (2016) and Wang, Zeng & Chen (2015).

To test the grasping ability of the fire ant, 10 workers varying in size from the treatment or control sub-colony were placed on an A4-sized paper. The paper was gently turned over 180 degrees after 10 s and held in place for 10 s. Workers falling from the paper was considered to have lost their grasping ability. The grasping ability was evaluated by grasping rate using the following formula: grasping rate (%) = (number of workers possessing grasping ability/total number of workers in test) × 100%. Each sub-colony from each treatment and control group was tested three times.

To test the walking speed of the fire ants, a tray with a fire ant sub-colony was connected to a forging arena (tray, L × W × H, 41.75 × 27.5 × 12 cm) by a wood bridge. The bridge consists of two 20 cm vertical segments and one 20 cm horizontal segment. The walking speed of the workers was measured by recording the time needed for an ant to walk across the 20 cm horizontal segment of the bridge. The method was described by Wang, Zeng & Chen (2015). A total of 25 workers were measured from each sub-colony.

### The effect of triflumezopyrim on the aggressiveness of *S. invicta*

In this experiment, we assessed the effect of sublethal doses of triflumezopyrim on the aggressive behavior of *S. invicta*. The experimental method was reflective of the method used by Pan, Lu & Wang (2017). The aggressiveness level of *S. invicta* was assessed when *S. invicta* individuals and colonies were confronted with another ant species, *Pheidole fervida*, at 56 days after treatment. Four *P. fervida* colonies were used.

*S. invicta* and *P. fervida* colonies were paired in inter specific individual or colony interaction tests. The inter specific interaction was quantified using a behavioral assay described by Pan, Lu & Wang (2017).

In inter specific individual aggression, interactions were scored and calculated as described by Rice & Silverman (2013) and Pan, Lu & Wang (2017). Four replicate experiments were performed for three pairs of colonies with each receiving a dose of triflumezopyrim. Each replicate involved different workers.

Inter specific colony aggression was evaluated using an assay described by Pan, Lu & Wang (2017). Specially, 30 workers varying in size from pairs of treated *S. invicta* and untreated *P. fervida* colonies were randomly chosen and introduced into a petri dish. Mortality rates of *S. invicta* were recorded after 3 h. Three replicates were performed for three pairs of colonies receiving a dose of triflumezopyrim at the same time. Each replicate involved different workers.

### Statistical analyses

All data were analyzed using Shapiro–Wilk and Levene’s tests for normal distribution and homogeneity of variances, respectively. If data were normally distributed and had similar variances, then the means of measured variables were compared by one-way analysis of variance (ANOVA). Significant ANOVA results were corrected for multiple comparisons using LSD’s method. Non-normally distributed data were analyzed using a non-parametric Kruskal–Wallis test to compare medians; differences significant at the 0.05 significance level were subjected to a Mann–Whitney test for pairwise comparisons. All statistical analyses were performed using SPSS version 18.0 (SPSS Inc., Chicago, IL, USA).

## Results

### Effect of triflumezopyrim on *S. invicta* colony growth

**Figure 1:**
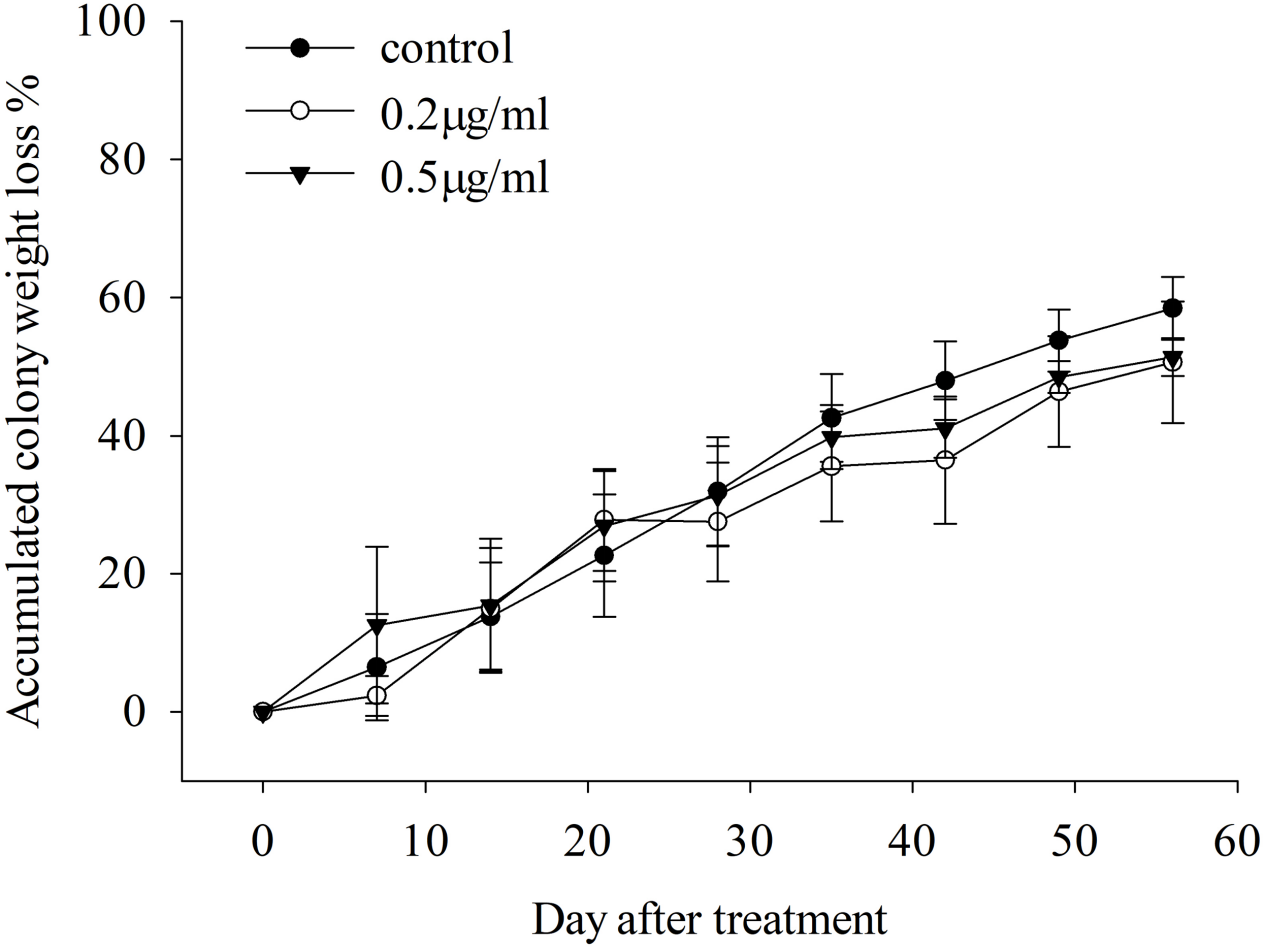
Accumulated colony weight loss (mean percentage ± SE) over 56 days after treatment of *Solenopsis invicta* with diﬀerent concentrations of triflumezopyrim.

There is no significant difference in the survival of *S. invicta* colonies among 0.5 μg/ml triflumezopyrim, 0.2 μg/ml triflumezopyrim, and the control (Fig. 1; day 7, LSD test, *F*_2, 9_ = 0.404, *P* = 0.679; day 14, LSD test, *F*_2, 9_ = 0.009, *P* = 0.991; day 21, LSD test, *F*_2, 9_ = 0.116, *P* = 0.892; day 28, LSD test, *F*_2, 9_ = 0.089, *P* = 0.916; day 35, LSD test, *F*_2, 9_ = 0.298, *P* = 0.749; day 42, LSD test, *F*_2, 9_ = 0.746, *P* = 0.501; day 49, LSD test, *F*_2, 9_ = 0.488, *P* = 0.629; day 56, LSD test, *F*_2, 9_ = 0.527, *P* = 0.608). After 56 days of treatment, the accumulated percentage weight losses were 51.40%, 50.65% and 58.46% in colonies treated with 0.5 μg/ml triflumezopyrim, 0.2 μg/ml triflumezopyrim, and controls, respectively.

### Effect of triflumezopyrim on *S. invicta* food consumption

There is no significant difference in sugar consumption between ants treated with 0.5 μg/ml triflumezopyrim, 0.2 μg/ml triflumezopyrim, and the control (Table 1; day 1, LSD test, *F*_2, 9_ = 0.946, *P* = 0.418; day 7, LSD test, *F*_2, 9_ = 0.673, *P* = 0.534; day 14, LSD test, *F*_2, 9_ = 0.322, *P* = 0.733; day 21, LSD test, *F*_2, 9_ = 1.396, *P* = 0.296; day 28, LSD test, *F*_2, 9_ = 2.129, *P* = 0.175; day 35, LSD test, *F*_2, 9_ = 0.283, *P* = 0.760; day 42, LSD test, *F*_2, 9_ = 1.472, *P* = 0.280; day 49, LSD test, *F*_2, 9_ = 1.768, *P* = 0.225; day 56, LSD test, *F*_2, 9_ = 0.782, *P* = 0.486).

**Table 1:** Sugar water consumption (mean ± SE) after 56 days treatment (number of colonies/treatment group = 4).

Day after treatment	Sugar water consumption (mg per gram workers)			Results of the one-way ANOVA	
	Control	0.2 μg/ml	0.5 μg/ml	*F*_2, 9_	*P*
1	149.7 ± 37.3a	214.6 ± 36.8a	195.5 ± 26.9a	0.946	0.418
7	145.4 ± 29.4a	120.5 ± 25.6a	102.6 ± 23.2a	0.673	0.534
14	139.7 ± 11.5a	152.1 ± 25.3a	130.8 ± 17.2a	0.322	0.733
21	153.5 ± 41.2a	185.7 ± 19.0a	112.6 ± 28.7a	1.396	0.296
28	135.8 ± 30.7a	77.0 ± 12.9a	96.9±12.3a	2.129	0.175
35	145.9 ± 17.3a	129.4 ± 22.3a	154.8 ± 31.0a	0.283	0.760
42	95.9 ± 16.7a	66.4 ± 7.9a	90.1 ± 12.5a	1.472	0.280
49	84.5 ± 8.4a	139.8 ± 34.8a	100.8 ± 9.5a	1.768	0.225
56	81.2 ± 10.8a	99.8 ± 11.9a	98.3 ± 12.2a	0.782	0.486

**Note:**

Same letter represents no significant difference within each observation period (*P* > 0.05).

Triflumezopyrim treatments do not have an impact on locust consumption (Table 2; day 1, LSD test, *F*_2, 9_ = 0.004, *P* = 0.996; day 7, LSD test, *F*_2, 9_ = 0.172, *P* = 0.845; day 14, LSD test, *F*_2, 9_ = 0.539, *P* = 0.601; day 21, LSD test, *F*_2, 9_ = 1.726, *P* = 0.232; day 28, LSD test, *F*_2, 9_ = 1.443, *P* = 0.286; day 35, LSD test, *F*_2,9_ = 3.691, *P* = 0.068; day 42, LSD test, *F*_2, 9_ = 0.728, *P* = 0.509; day 49, LSD test, *F*_2, 9_ = 1.940, *P* = 0.199; day 56, LSD test, *F*_2, 9_ = 0.089, *P* = 0.915).

**Table 2:** Locust consumption (mean ± SE) after 56 days treatment (number of colonies/treatment group = 4).

Day after treatment	Sugar water consumption (mg per gram workers)			Results of the one-way ANOVA	
	Control	0.2 μg/ml	0.5 μg/ml	*F*_2, 9_	*P*
1	71.4 ± 47.0a	76.5 ± 33.3a	72.6 ± 47.6a	0.004	0.996
7	33.5 ± 33.4a	41.9 ± 18.6a	20.0 ± 26.1a	0.172	0.845
14	17.5 ± 13.9a	38.0 ± 16.8a	30.3 ± 10.9a	0.539	0.601
21	30.6 ± 8.4a	63.6 ± 20.2a	67.1 ± 15.1a	1.726	0.232
28	23.8 ± 7.5a	33.9 ± 5.9a	15.0 ± 9.8a	1.443	0.286
35	39.6 ± 16.6a	67.8 ± 18.0a	102.8 ± 14.5a	3.691	0.068
42	−15.1 ± 19.9a	10.3 ± 23.1a	22.4 ± 24.1a	0.728	0.509
49	−1.6 ± 12.1a	49.6 ± 29.1a	61.6 ± 27.3a	1.940	0.199
56	36.6 ± 17.4a	40.4 ± 28.2a	52.8 ± 36.0a	0.089	0.915

**Note:**

Same letter represents no significant difference within each observation period (*P* > 0.05).

### Effect of triflumezopyrim on *S. invicta* activity behavior

After 56 days of treatment the grasping rate of workers was 88.67 ± 2.14%, 90.33 ± 1.67% and 71.67 ± 5.13% in 0.5 μg/ml triflumezopyrim, 0.2 μg/ml triflumezopyrim, and the control, respectively. The grasping rate of workers in the triflumezopyrim treatment is higher than that of the control (0.5 μg/ml triflumezopyrim and control, Mann–Whitney test, *U* = 27, *P* = 0.009; 0.2 μg/ml triflumezopyrim and control, Mann–Whitney test, *U* = 18, *P* = 0.002).

After 56 days of treatment the walking speed of workers was 9.50 ± 0.22 mm/s, 9.39 ± 0.23 mm/s and 9.41 ± 0.38 mm/s in 0.5 μg/ml triflumezopyrim, 0.2 μg/ml triflumezopyrim, and control, respectively. The walking speed of workers in 0.5 μg/ml triflumezopyrim treatment was higher than that of the control (0.5 μg/ml triflumezopyrim and control, Mann–Whitney test, *U* = 4044.5, *P* = 0.02), and the walking speed of workers in the 0.2 μg/ml triflumezopyrim treatment is not significantly different from either that in the 0.5 μg/ml triflumezopyrim or the control (0.2 μg/ml triflumezopyrim and control, Mann–Whitney test, *U* = 4199.5, *P* = 0.050; 0.2 μg/ml triflumezopyrim and 0.5 μg/ml triflumezopyrim, Mann–Whitney test, *U* = 4733.0, *P* = 0.514).

### Effect of triflumezopyrim on *S. invicta* aggressiveness

After 56 days of treatment the aggressiveness indices of *S. invicta* workers was 1.58 ± 0.28, 1.31 ± 0.29 and 0.47 ± 0.20 in 0.5 μg/ml triflumezopyrim, 0.2 μg/ml triflumezopyrim, and the control, respectively. The aggressiveness index of *S. invicta* was significantly increased by triflumezopyrim treatment (0.5 μg/ml triflumezopyrim and control, Mann–Whitney test, *U* = 57.5, *P* = 0.004; 0.2 μg/ml triflumezopyrim and control, Mann–Whitney test, *U* = 75.5, *P* = 0.030).

In the group aggression experiment, the mortality of *S. invicta* workers was 6.33 ± 1.51%, 6.67 ± 1.80%, 7.33 ± 2.14% in 0.5 μg/ml triflumezopyrim, 0.2 μg/ml triflumezopyrim, and control after 56 days of treatment, respectively; there is no significant difference among triflumezopyrim treatments and the control (LSD test, *F*_2, 33_ = 0.77, *P* = 0.926).

## Discussion

The negative impact of neonicotinoids on non-target organisms is a global concern. As a novel neonicotinoid, the impact of triflumezopyrim on non-target insects deserves attention. Zhu et al. (2018) found that 62.5 μg·a.i/ml triflumezopyrim does not negatively impact the parasitic wasp *Anagrus nilaparvatae* after 30 min of exposure. Our study showed that 0.2 μg/ml and 0.5 μg/ml triflumezopyrim, which are near the tolerance levels for the residue of triflumezopyrim in rice grains and hulls, does not have a negative effect on the colony growth and food consumption (sugar water and locusts) of fire ants after 56 day of exposure. Although the 0.2 μg/ml and 0.5 μg/ml triflumezopyrim treatments increased the grasping ability and individual aggressiveness of fire ant workers, and 0.5 μg/ml triflumezopyrim treatment increased the walking speed of fire ants, triflumezopyrim treatment did not impact the mortality of fire ant workers in the group aggression experiment.

Rondeau et al. (2014) suggested that testing for the chronic effects of pesticides should be extended to 30 days or more for social insects. The estimated average life span of minor, medium, and major workers is 45 days, 75 days and 135 days, respectively (Calabi & Porter, 1989). Our experiments were conducted for 56 days in light of the above mentioned factors. We used three experimental parameters to investigate the impact of tolerances for the residues of triflumezopyrim exposure on fire ant colony growth, food consumption and activity. The three parameters were chosen because they are related to the fitness of the fire ant colony. For example, walking speed and aggressiveness is involved in competition for food, and the protection and expansion of territory, which are indicators of competitiveness in ants (Holway, 1999).

Our results showed that the treatments of 0.2 μg/ml and 0.5 μg/ml triflumezopyrim increased the grasping ability and aggressiveness of the individual worker and 0.5 μg/ml triflumezopyrim increased the walking speed of the fire ant. Other neonicotinoids have a similar effect. For example, thiamethoxam at 108.1 ng/g exposure caused a short-term locomotor hyperactivity of the carabid beetle *Platynus assimilis* (Tooming et al., 2017). The lower concentration of imidacloprid, such as 10 ng or 1.25 ng/insect treatment, resulted in a higher locomotor activity of the treated insect (Galvanho et al., 2013; Lambin et al., 2001). De França et al. (2017) indicated that insect behavior changes with sublethal doses of pesticides, which may indirectly influence species biology and fitness. However, behavior changes resulting in fire ant fitness for colony growth and group aggression was not impacted based on our data.

Registration information of triflumezopyrim is submitted to many key markets of the world and is now registered for sale in China (http://news.agropages.com/News/NewsDetail---19109.htm). Our study revealed that there is no negative impact of 0.5 μg/ml triflumezopyrim exposure on the colony growth and food consumption of the fire ant. Further studies are needed to determine the conditions under which triflumezopyrim may have a detrimental impact on ants. Our results provide information for the future use of this insecticide on crops in terms of regulations and policy decisions.

## Conclusions

We found triflumezopyrim, a novel neonicotinoid, did not cause any negative impact on the fitness of the fire ant *S. invicta* at 0.2 μg/ml and 0.5 μg/ml after 56 days of observation. Neither colony growth nor food consumption was influenced by 0.2 μg/ml and 0.5 μg/ml triflumezopyrim. The probability of survival of *S. invicta* was not impacted by triflumezopyrim treatments in aggressive group encounters, although 0.5 μg/ml and 0.2 μg/ml triflumezopyrim increased the individual aggressive index, and 0.5 μg/ml triflumezopyrim increased the moving speed and grasping ability of *S. invicta*. The results imply that the maximum residue limit of triflumezopyrim may have no impact on other non-target ants.

## Supplemental Information

10.7717/peerj.8241/supp-1 Supplemental Information 1 Raw Data of dose residues of triflumezopyrim exposure have negative effect on ant?

## Additional Information and Declarations

### Data Availability

The following information was supplied regarding data availability: Raw data for this manuscript is available in a Supplemental File.

## References

[ref-1] Barbieri RF, Lester PJ, Miller AS, Ryan KG (2013). A neurotoxic pesticide changes the outcome of aggressive interactions between native and invasive ants. Proceedings of the Royal Society B: Biological Sciences.

[ref-2] Butcherine P, Benkendorff K, Kelaher B, Barkla BJ (2019). The risk of neonicotinoid exposure to shrimp aquaculture. Chemosphere.

[ref-3] Calabi P, Porter SD (1989). Worker longevity in the fire ant *Solenopsis invicta*: ergonomic considerations of correlations between temperature, size and metabolic rates. Journal of Insect Physiology.

[ref-4] Cordova D, Benner EA, Schroeder ME, Holyoke CW, Zhang W, Pahutski TF, Leighty RM, Vincent DR, Hamm JC (2016). Mode of action of triflumezopyrim: a novel mesoionic insecticide which inhibits the nicotinic acetylcholine receptor. Insect Biochemistry and Molecular Biology.

[ref-5] De França SM, Breda MO, Barbosa DRS, Araujo AMN, Guedes CA, Shields VDC (2017). The sublethal effects of insecticides in insects. Biological Control of Pest and Vector Insects.

[ref-6] Galvanho JP, Carrera MP, Moreira DDO, Erthal M, Silva CP, Samuels RI (2013). Imidacloprid inhibits behavioral defences of the leaf-cutting ant *Acromyrmex subterraneus subterraneus* (Hymenoptera: Formicidae). Journal of Insect Behavior.

[ref-7] Harvey CT, Eubanks MD (2004). Effect of habitat complexity on biological control by the red imported fire ant (Hymenoptera: Formicidae) in collards. Biological Control.

[ref-8] Hesselbach H, Scheiner R (2019). The novel pesticide flupyradifurone (Sivanto) affects honeybee motor abilities. Ecotoxicology.

[ref-9] Hladik ML, Main AR, Goulson D (2018). Environmental risks and challenges associated with neonicotinoid insecticides. Environmental Science & Technology.

[ref-10] Holway DA (1999). Competitive mechanisms underlying the displacement of native ants by the invasive argentine ant. Ecology.

[ref-11] Hölldobler B, Wilson EO (1990). The ants.

[ref-12] Huang SQ, Fu JT, Wang K, Xu HH, Zhang ZX (2016). Insecticidal activity of the methanol extract of *Pronephrium megacuspe* (Thelypteridaceae) and its active component on *Solenopsis invicta* (Hymenoptera: Formicidae). Florida Entomologist.

[ref-13] Ihara M, Matsuda K (2018). Neonicotinoids: molecular mechanisms of action, insights into resistance and impact on pollinators. Current Opinion in Insect Science.

[ref-14] Lambin M, Armengaud C, Raymond S, Gauthier M (2001). Imidacloprid-induced facilitation of the proboscis extension reflex habituation in the honeybee. Archives of Insect Biochemistry and Physiology.

[ref-15] Pan F, Lu Y, Wang L (2017). Toxicity and sublethal effects of sulfoxaflor on the red imported fire ant, *Solenopsis invicta*. Ecotoxicology and Environmental Safety.

[ref-16] Pisa LW, Amaral-Rogers V, Belzunces LP, Bonmatin JM, Downs CA, Goulson D, Kreutzweiser DP, Krupke C, Liess M, McField M, Morrissey CA, Noome DA, Settele J, Simon-Delso N, Stark JD, Van Der Sluijs JP, Van Dyck H, Wiemers M (2015). Effects of neonicotinoids and fipronil on non-target invertebrates. Environmental Science and Pollution Research.

[ref-18] Rice ES, Silverman J (2013). Submissive behaviour and habituation facilitate entry into habitat occupied by an invasive ant. Animal Behaviour.

[ref-19] Rondeau G, Sánchez-Bayo F, Tennekes HA, Decourtye A, Ramírez-Romero R, Desneux N (2014). Delayed and time-cumulative toxicity of imidacloprid in bees, ants and termites. Scientific Reports.

[ref-20] Shoemaker D, Ascunce MS (2010). A new method for distinguishing colony social forms of the fire ant, *Solenopsis invicta*. Journal of Insect Science.

[ref-21] Siviter H, Scott A, Pasquier G, Pull CD, Brown MJF, Leadbeater E (2019). No evidence for negative impacts of acute sulfoxaflor exposure on bee olfactory conditioning or working memory. PeerJ.

[ref-22] Tan K, Wang C, Dong S, Li X, Nieh JC (2017). The pesticide flupyradifurone impairs olfactory learning in Asian honey bees (*Apis cerana*) exposed as larvae or as adults. Scientific Reports.

[ref-23] Thiel S, Köhler H-R (2016). A sublethal imidacloprid concentration alters foraging and competition behaviour of ants. Ecotoxicology.

[ref-24] Tooming E, Merivee E, Must A, Merivee M-I, Sibul I, Nurme K, Williams IH (2017). Behavioural effects of the neonicotinoid insecticide thiamethoxam on the predatory insect *Platynus assimilis*. Ecotoxicology.

[ref-25] Toro ID, Ribbons RR, Pelini SL (2012). The little things that run the world revisited: a review of ant-mediated ecosystem services and disservices (Hymenoptera: Formicidae). Myrmecological News.

[ref-26] Tosi S, Nieh JC (2019). Lethal and sublethal synergistic effects of a new systemic pesticide, flupyradifurone (Sivanto^®^), on honeybees. Proceedings of the Royal Society B: Biological Sciences.

[ref-27] Vinson SB (2013). Impact of the invasion of the imported fire ant. Insect Science.

[ref-28] Vogt JT, Grantham RA, Smith WA, Arnold DC (2001). Prey of the red imported fire ant (Hymenoptera: Formicidae) in Oklahoma peanuts. Environmental Entomology.

[ref-29] Wang X, Anadon A, Wu Q, Qiao F, Ares I, Martinez-Larranaga M-R, Yuan Z, Martinez M-A (2018). Mechanism of neonicotinoid toxicity: impact on oxidative stress and metabolism. Annual Review of Pharmacology and Toxicology.

[ref-30] Wang L, Wang Z, Zeng L, Lu Y (2016). Red imported fire ant invasion reduces the populations of two banana insect pests in South China. Sociobiology.

[ref-31] Wang L, Zeng L, Chen J (2015). Sublethal effect of imidacloprid on *Solenopsis invicta* (Hymenoptera: Formicidae) feeding, digging, and foraging behavior. Environmental Entomology.

[ref-32] Wurm Y, Wang J, Riba-Grognuz O, Corona M, Nygaard S, Hunt BG, Ingram KK, Falquet L, Nipitwattanaphon M, Gotzek D, Dijkstra MB, Oettler J, Comtesse F, Shih C-J, Wu W-J, Yang C-C, Thomas J, Beaudoing E, Pradervand S, Flegel V, Cook ED, Fabbretti R, Stockinger H, Long L, Farmerie WG, Oakey J, Boomsma JJ, Pamilo P, Yi SV, Heinze J, Goodisman MAD, Farinelli L, Harshman K, Hulo N, Cerutti L, Xenarios I, Shoemaker D, Keller L (2011). The genome of the fire ant *Solenopsis invicta*. Proceedings of the National Academy of Sciences of the United States of America.

[ref-33] Zhang W (2017). Mesoionic pyrido[1,2-*a*]pyrimidinone insecticides: from discovery to triflumezopyrim and dicloromezotiaz. Accounts of Chemical Research.

[ref-34] Zhang W, Holyoke CW, Pahutski TF, Lahm GP, Barry JD, Cordova D, Leighty RM, Singh V, Vicent DR, Tong M-HT, Hughes KA, McCann SF, Henry YT, Xu M, Briddell TA (2017). Mesoionic pyrido[1,2-*a*]pyrimidinones: discovery of triflumezopyrim as a potent hopper insecticide 1. Bioorganic & Medicinal Chemistry Letters.

[ref-35] Zhu J, Li Y, Jiang H, Liu C, Lu W, Dai W, Xu J, Liu F (2018). Selective toxicity of the mesoionic insecticide, triflumezopyrim, to rice planthoppers and beneficial arthropods. Ecotoxicology.

